# Waging war against pancreatic cancer: an interview with David Tuveson

**DOI:** 10.1242/dmm.029975

**Published:** 2017-04-01

**Authors:** David Tuveson

**Affiliations:** Cold Spring Harbor Cancer Center, Cold Spring Harbor, NY 11724, USA

## Abstract

David Tuveson, Director of the Cancer Center at Cold Spring Harbor Laboratory, is a clinician-scientist with a longstanding interest in understanding and treating pancreatic cancer. Since developing the first mouse model of pancreatic cancer in 2002, the Tuveson lab has made a series of discoveries that shed light on the molecular drivers of this disease and provide promising therapeutic avenues for a malignancy that is notoriously challenging to treat. In collaboration with Hans Clevers, David developed the first pancreatic cancer organoids, which revolutionized the field by providing a powerful model system for basic discoveries and advancement of personalized medicine. Here, David talks to Ross Cagan about his path from chemistry student to world-renowned oncologist, highlighting how his colleagues, mentors and patient interactions shaped his research interests and unique approach to scientific discovery. As well as discussing the story behind some of his breakthroughs, he provides tips on running a lab and succeeding in or outside academia.

## Abstract (precis)

**Summary:** David Tuveson describes some of the key moments and breakthroughs in his remarkable career, and the path that he's followed in his quest for a cure for pancreatic cancer.


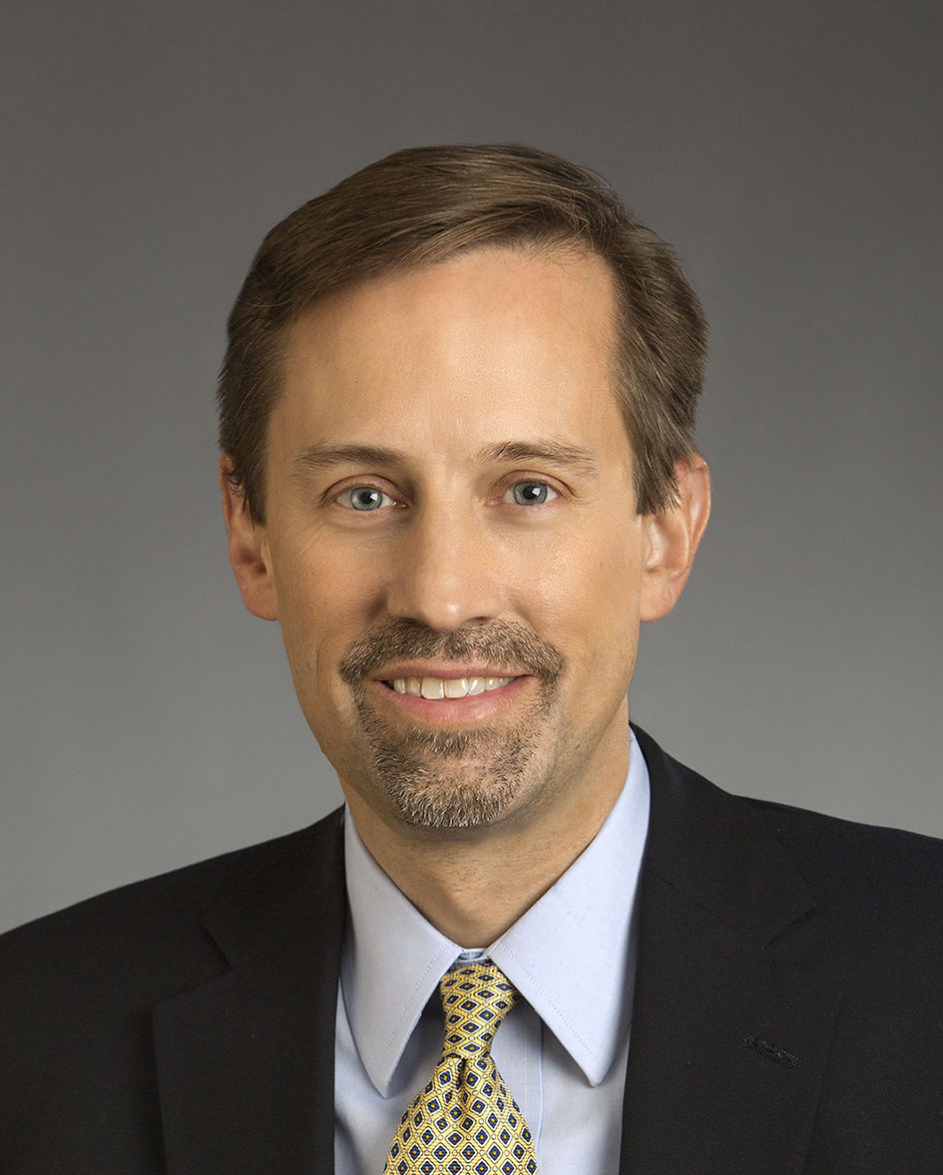


David Tuveson was born in Chicago, in 1966. He received his BSc in Chemistry from Massachusetts Institute of Technology (M.I.T.) in 1987, and after completing his MD PhD at Johns Hopkins School of Medicine in 1994, he returned to Boston to undertake an Internal Medicine Residency at Brigham & Women's Hospital. Settling on oncology as his specialist area, David pursued a fellowship at the Dana-Farber Cancer Institute between 1997 and 2000. As part of his post-doctoral training, David worked with George Demetri to develop c-Kit inhibitors as a treatment for gastrointestinal stromal tumors, and also created the first KRAS-dependent lung cancer mouse models with Tyler Jackson at MIT. He became an Assistant Professor at the University of Pennsylvania in 2002, and it was here that he succeeded in developing the first mouse models of ductal pancreatic cancer. In 2006, he moved to the UK where he founded and directed the Cambridge Pancreatic Cancer Centre. During his years in Cambridge, he brought to light a key role for stromal interactions in impeding drug delivery into pancreatic tumors, and these findings formed the basis of clinical trials for this particularly aggressive type of cancer. He returned to the USA in 2012 to direct the Cancer Therapeutics Initiative at Cold Spring Harbor Laboratory (CSHL), and to date remains dedicated to straddling the clinic and lab to improve the outcome for pancreatic cancer patients. His lab uses mouse models to explore the molecular mechanisms that drive pancreatic cancer, and in 2012 they partnered with the Clevers’ lab at the Hubrecht Institute to develop pancreatic cancer organoids – three-dimensional tissue-based models of the disease. David was recently appointed as Director of the National Cancer Institute (NCI)-designated Cancer Center at CSHL, and continues to practice medical oncology with an adjunct appointment at Memorial-Sloan Kettering and a clinical affiliation with Northwell Health. He has been honoured with several awards, including the Rita Allen Foundation Scholar Award, the Waldenstrom Award and the Hamdan Award.

**Let's start with your background. Have you always been interested in science?**

My parents played an important role. I would go outside with a net and catch things that fly or swim and bring them home to try to identify them – my parents really encouraged this. Growing up, my curiosity was mostly embedded in nature, and I loved exploring the myriad of shapes, sizes, colors and patterns that occur naturally. I was fascinated with the outdoor world, and I suspect today this experience has been replaced by sitting on a couch for many youngsters. At school, I was able to channel my curiosity towards understanding and learning new things. Then, when I got to university, I was surrounded by people much smarter than I was and that was probably the shifting event. I've always been a good observer, but my peers were better at quantifying things and solving problems. So I decided to get better at the quantitative stuff. I majored in Chemistry at MIT, not necessarily because I loved it, but because it was hard. My mentors at MIT were amazing. My freshman advisor was Robert Langer; a brilliant, clever, creative guy who I've kept in touch with over the years. (Karl) Barry Sharpless was then my advisor in chemistry, and he is also incredibly creative; he worked on developing new ways to synthesize compounds and he won a Nobel Prize in Chemistry for his work. Barry is the person who helped me to channel my interest in chemistry towards solving problems in life sciences, and he got me to think about going to medical school, which is ultimately what I chose to do.

**Can you tell us a bit about your experiences and mentors at Johns Hopkins?**

Medical school was one of the most profound experiences of my life. The coursework in the beginning was a little bit slow, but it picked up as soon as patients got involved. For my research project, I wanted to work on the science of sleep, but I struggled to find an advisor. This was before there were any genes on circadian rhythm cloned, and people wanted to work on areas that were maybe more focused, such as olfaction. Instead, I ended up working on the immune system. This was a part of physiology that was ‘coming of age’ thanks to advances in molecular biology, and I was lucky enough to do a rotation in Doug Fearon's lab. Doug is an immunologist and he had moved to John Hopkins from Harvard where he had done his seminal work on the complement system. Working with Doug gave me my first proper laboratory exposure and experience of ‘deep science’, and I loved it. He is very smart, very serious and he was a great role model.

 “Cancer patients reminded me of experiments where you didn't know if you were going in the right direction, you didn't know what was going to happen, you just knew you cared.”

**What inspired your interest in oncology?**

After finishing my MD PhD, I decided to do an Internal Medicine Residency in Boston. I found practicing as a doctor easier than doing research because you can see the ‘finish line’ – you can actually help people in a quick period of time, though not always. The ‘not always’ patients oftentimes had cancer. Cancer patients reminded me of experiments where you didn't know if you were going in the right direction, you didn't know what was going to happen, you just knew you cared. It disturbed me that we had so little to offer cancer patients, which was a driving force behind my decision to go into oncology. It was between this or cardiology, which I still think is a lot more fun because once you learn how to fix a person's heart you can put them back in their office or on their golf course; in cancer we don't have any of those kind of ‘baby delivery’ moments. So my decision wasn't based on science so much as a desire to help patients.

**Why did you focus on pancreatic cancer? What makes this type of cancer such a ‘rugged opponent’?**

Going back a little, when I was a house officer at the Brigham in 1994, I thought I was going to end up being an HIV doctor, because at the time there was nothing we could offer these patients. But as it turned out, that was the year that combination antiretroviral therapy came out, and the patients went from dying in front of our eyes because of opportunistic infections, to getting better. Pancreatic cancer was a bigger challenge. At the time, targeted therapy didn't exist and the patients that I saw never got better, and nobody was interested. I ran around Boston trying to find *somebody* to talk to about the disease, and was lucky to get steered towards a new faculty member at MIT - Tyler Jacks. I met Tyler, and he wasn't that thrilled with the idea of having a medically trained post-doc in his lab, but I think the fact that I had research experience persuaded him. So in 1996, I became his first medical post-doc. I worked on trying to create a model of pancreatic cancer, and I hadn't succeeded even by 2002 when I got recruited by UPenn. After I arrived at UPenn, I bred the mice that I made at MIT together, and within the first few months it was very clear they were showing pancreatic pathology – the model itself was thus born at UPenn, but gestated at MIT. Tyler was very generous; he allowed me to take the whole project with me and was very supportive of my career. This is how I try to be with all my post-docs. I tell them, ‘come and work on your problem, and I cannot promise you that you'll solve it but I can promise you that I'm going to support you, come hell or high water’. I also tell my lab that my job as a pancreatic cancer researcher – and theirs – is to put me out of work. For example, the characterization of the first mouse models was massively accelerated by a long-time colleague and friend who joined our lab as an instructor a few months after I started at Penn, Sunil Hingorani. Sunil was so productive that he was able to get his own independent faculty position within three years! Sunil's own lab has accelerated the field greatly, and I am always looking for the next Sunil. I've been working on pancreatic cancer for 20 years now, and I would have to say that all I've done is to help the field understand the problem better; no solving has happened, though we're getting closer.

 “I've been working on pancreatic cancer for 20 years now, and I would have to say that all I've done is to help the field understand the problem better; no solving has happened, though we're getting closer.”

**How has your time in the lab changed who you are as a doctor?**

I don't spend a lot of my time seeing patients any more, and spend more time on the research side. I think that the lab offers the best hope for patients; for example, by identifying better therapies or developing a test that can detect pancreatic cancer early. These are two key challenges that we are working on addressing in our lab. I feel lucky that I have the opportunity to do both medicine and research - because I spent so many years learning to become a physician, and really enjoyed it, I can speak both ‘languages’ and I have a feel for what we need to do better to help patients. Not everything we have worked on in the lab has borne fruit in the clinic yet, but everything we have worked on has helped us to understand the clinical problem better and brought us a step closer. What I do is very exciting, but it's also very sobering. The time I spend with patients, I'm constantly reminded we have to move faster and move smarter to come up with things that don't just equal great papers but could actually impact patients.

**How has the pancreatic cancer community changed over 20 years?**

Well there are now thousands of brilliant researchers working on pancreatic cancer, which is great. The tools have also changed. We now have models that we can use to follow cancer progression. These models are important because patients get sick so fast that you can't really study them – it's not possible, it's not ethical – you just focus on alleviating their suffering. When patients are that sick, they also don't qualify for clinical trials and so you don't learn scientifically what they're going through. I think the science that we have developed – my lab and many of the people that have come into the field – offers a ray of sunlight into this dark room. I'm not a chemist any more, but there are chemists now working on the disease as well as geneticists. There are people trying harder than ever before to cure this disease.

 “What I do is very exciting, but it's also very sobering. The time I spend with patients, I'm constantly reminded we have to move faster and move smarter to come up with things that don't just equal great papers but could actually impact patients.”

**Talking about trying – a few years ago you ran a trial for a stromal hedgehog inhibitor, which failed to slow progression of pancreatic cancer. What was the background behind this trial and what did you learn from the experience?**

The experiments were done by a terrific post-doc, Ken Olive, back when my lab was in Cambridge [UK]. The problem with pancreatic cancer is that it's pretty insensitive to chemotherapy. We started testing the efficacy of gemcitabine in GEMMs [genetically engineered mouse models] of pancreatic cancer, and found that it didn't work well. Surprisingly, gemcitabine worked much better when we prepared subcutaneous transplantation mouse cancer models, which are used by most researchers and corporations. It also didn't matter if the subcutaneous tumors were of mouse or human origin. We soon came to realize that impaired drug delivery was behind the chemoresistance of GEMMs, specifically because the vasculature was poor compared with transplanted tumors. We confirmed this independently by showing that human PDA [pancreatic ductal adenocarcinoma] samples showed hypovascularity. We decided to take it further – since nobody would publish this observation alone – by looking at whether disrupting the stroma might positively impact tumor vasculature and improve drug delivery. The first compound we looked at was an inhibitor of Smoothened (Smo) in the Hedgehog pathway. It turned out that Smo inhibition depleted stromal abundance in the pancreatic tumors in our mouse model, and the number of blood vessels sprouted way up. When we administered the Smo inhibitor at the same time as gemcitabine, we could show that the drug got in the tumour and killed cancer cells, leading to tumor shrinkage in some of the mice. The animals lived for a couple of weeks longer, and then they all died. We looked in the tumours and the number of blood vessels had gone back down again and the stroma was different.

Based on these data, we hypothesized that we could fix the perfusion problem to improve drug delivery. The company that we worked with, Infinity, was very enthusiastic about these results and did clinical trials for the Smo inhibitor right away. The Phase I trial showed that it wasn't dangerous, and it moved into a randomized Phase 2 trial in which patients underwent chemotherapy, also using gemcitabine, with and without the Smo inhibitor. Although this trial still hasn't been formally published, they realized that the patients who had received both drugs actually died faster, so the trial was halted. When people called me to talk about what had happened, the first thing I asked was ‘what do the samples show?’, and they said, ‘what samples?’ I became very frustrated not only at the outcome but in the way that trials are designed, without advice from scientists and without looking at the findings scientifically. Ken, who by now had set up his own lab studying the hedgehog signaling pathway in pancreatic cancer, was obviously troubled by this too, and so he went back to mice and realized that if you *only* gave a Smo inhibitor, you would promote a very aggressive form of pancreatic cancer and the animals died with rampant cachexia, even though the tumors were smaller. His colleague at UPenn, Ben Stanger, confirmed this genetically. When I talked to the clinicians who did the trial, they told me that the patients got worse on both drugs but not because the cancer was growing. Patients got worse because their body collapsed. Out of this disappointment came this fascinating observation that drove us to go back to look at what the pancreas tumour is really made out of. We developed a new organoid model of pancreas cancer with Hans Clevers when we moved back to the US, and have made some headway using this model to understand what's going on at the cellular level.

Despite the failure of the trial, I firmly believe that our pre-clinical experiments with mice put us on the right track by showing that the barrier of drug delivery is what we need to work on, and we need to study it scientifically to make sure that we don't cause other physiological problems by interfering. It's not the same as unplugging the sink in your kitchen: if you're going to alter biology in the non-neoplastic compartment, you need to remember that this compartment is highly adaptable.

 “Identify the problem, understand it at least to the level where you have therapies that can help that patient, and then let them return to their families…*That's* where I want cancer to be, and it's not there yet.”

**Is this what motivated you to move over to Cold Spring Harbor?**

My plan was to set up a preclinical facility that did very deep science using the mouse models and organoid models, but simultaneously establish a carbon copy of that in a human clinic. As you know, Cold Spring Harbor is a basic science campus with no hospital nearby and no medical students running around, so we have established a partnership with a very large healthcare provider in Long Island called Northwell Health. The idea behind this relationship was that we could build a Phase 0/Phase I experimental therapeutic facility, where proof-of-concept, proof-of-mechanism clinical trials can be performed; where patients can remain from 24 hours to several days undergoing multiple biopsies, pharmacological monitoring, imaging and so forth to answer the question: is the scientific hypothesis correct? We've hired a great clinician scientist to run the facility, Robert Maki, who was my senior resident when I was an intern at the Brigham.

My vision for the future of cancer research is that the patient comes into your hospital, you get a biopsy, you perform molecular diagnostics, you perform pharmacological testing either on a piece of the tumor itself or an organoid generated from the tumor, and then you invite the patient to stay with you until you have a therapy that you know is helping them. Identify the problem, understand it at least to the level where you have therapies that can help that patient, and then let them return to their families. This is how the cardiology field works: if you have chest pain and they're pretty sure you have a blockage in your artery, you're not going home until they know where that blockage is and have fixed it! *That's* where I want cancer to be, and it's not there yet.

“Organoids are crazy interesting, and when I see other people in the pancreatic cancer field I tell them, you should stop what you're doing and work on these because it's the faster way of studying this disease.”

**You've mentioned organoids, which I know you're pretty involved in with Hans Clevers. What are your plans for organoids of pancreatic cancer?**

Organoids are a really terrific model of a patient's tumour that you generate from tissue that is either removed at the time of surgery or when they get a small needle biopsy. Culturing the tissue and observing an outgrowth of it is usually successful and when you have the cells, you can perform molecular diagnostics of any type. With a patient-derived organoid, you can sequence the exome and the RNA, and you can perform drug testing, which I call ‘pharmacotyping’, where you're evaluating compounds that by themselves or in combination show potency against the cells. A major goal of our lab is to work towards being able to use organoids to choose therapies that will work for an individual patient – personalized medicine.

Organoids could be made moot by implantable microdevices for drug delivery into tumors, developed by Bob Langer. These devices are the size of a pencil lead and contain reservoirs that release microdoses of different drugs; the device can be injected into the tumor to deliver drugs, and can then be carefully dissected out and analyzed to gain insight into the sensitivity of cancer cells to different anticancer agents. Bob and I are kind of engaged in a friendly contest to see whether organoids or microdosing devices are going to come out on top. I suspect that both approaches will be important for pharmacotyping cancer patients in the future.

From the science side, we use organoids to discover things about pancreatic cancer. They're great models, probably the best that I know of to rapidly discover new things about cancer because you can grow normal tissue as well as malignant tissue. So, from the same patient you can do a comparison easily to find out what's different in the tumor. Organoids are crazy interesting, and when I see other people in the pancreatic cancer field I tell them, you should stop what you're doing and work on these because it's the faster way of studying this disease.

**Let's move a little bit away from the science. You've bounced back and forth a bit between the UK and the US; what are the differences you've seen between the approaches used on both sides?**

Scientists in the UK and the US are similar in most regards but there are some differences. In the UK, people tend to think before they jump, they tend to talk to each other about their ideas a lot more that we do here in America, they meet twice a day to discuss things – often during tea breaks – and they tend to experiment less. I'd say in America we do ten experiments to every two that are performed in the UK. But we tend to do a lot of experiments that don't work. I would say that the two experiments they do in the UK don't necessarily work either, but probably one does. I feel that this is a cultural difference: the British tend to spend less money doing things but spend more time thinking about them. It doesn't mean they're smarter, it's just their approach to life. I found it refreshing, I never stopped to have tea at the beginning of the day or the end of the day but a lot of people who worked in my lab did. I would eat my lunch in my office while working but I probably loosened up a little bit by the end of the six years that I was there and spent more time talking to people. I really loved my time there, my colleagues were spectacular and I did some terrific science there. I hope that the people who read this think about doing some research abroad, out of their comfort zone.

“I tell my post-docs, roll your sleeves up and get your hands dirty.”

**You've trained a lot of people who have gone on to have some success. What type of mentor are you? What are your tips for running a successful lab?**

First and foremost, I try to identify people who have an internal passion about solving problems, because you can't make people passionate, though you can make them smarter. Once we've worked out what they're excited about, I help with the development of the idea and how they're going to go about it, but it's very important that they have ownership. As a mentor, I explain that I don't have all the answers but I have an approach that seems to bear fruit and if they're passionate about solving a problem, then I will help them in any way I can. I've developed a mnemonic for what I think is the ideal approach to science – PHENOM, which stands for P is problem, H is hypothesis, E is experimental methods, N is notebook, O is oration, M is meeting. I tell them, figure out what problem you want to solve, develop your own hypotheses and always use multiple experimental approaches, because if your hypothesis is right, they will support it and if your hypothesis is partially right, they will at some point show you why you're wrong. I also tell them you have to love to work. My work ethic comes from upbringing; I grew up catching butterflies and snakes and everything else, rather than sitting and wondering if one day I'll catch one. So I tell my post-docs, roll your sleeves up and get your hands dirty. Make collaborations with people who can help you when you have no clue what to do, but don't stop doing experiments because you need data that you can interpret and analyze. That's the N of PHENOM: notebook. Figure out what you've got, write it down and think about it. It's common to get stuck when we have no clue what the data's telling us and that's when you should become British, when you need to talk to people. Scientists love talking about data. We then come to the O, which refers to oration – this is where I emphasize the importance of learning to give a talk, and M is for meeting; to meet colleagues and let them help you. My approach might not work for everybody, but I love what I do and I wouldn't trade it in for anything, so I guess I primarily lead by example.

“You have to be resilient to be a scientist. You have to enjoy the ride, enjoy working hard, and recognize not everything has to work the first time.”

**A lot of people coming out of labs now are concerned about the future of research. Do you have any advice to them?**

When I was their age the fields of molecular biology and cancer research, and the biotech sector were much smaller so there was less competition. Now it is harder to win a grant or publish papers. Nine out ten times you send a paper to a high-impact journal, it doesn't get accepted either. You have to be resilient to be a scientist. You have to enjoy the ride, enjoy working hard and recognize not everything has to work the first time. If you're passionate about what you're doing and it's original, you're going to be fine*.* But I think that we as professors need to be more vocal that our path is not for everyone, it's for the people who will work hard and not get disappointed when their paper doesn't get accepted the first time. Some people think we train too many PhD students and too many post-docs, but I don't agree with this at all: I think we need educated people and critical thinkers to fill all sectors in our society. Somebody I trained was supposed to go to medical school, but he became a high-school chemistry teacher. He realized his passion was to teach because he was my best darn tech, teaching everybody else how to do things! I think we need to encourage our trainees to find what they really love doing. Many of my post-docs love science because they feel the joy of performing experiments, but I have also trained some great post-docs who write well, think well and speak well, but are just not experimentalists. So their path should be something else, since they have different skills and passions. This doesn't mean they have failed but rather that they recognize their real strengths.

I also think we need to stop spreading this concept of doom and gloom in the job market and recognize the tremendous opportunities we now have in biomedical science that we didn't in the past. In cancer, the number of things that we can do to help patients today is *huge* compared to even ten years ago. The field of cancer immunology, for example, came out of nowhere. Look at it now: it's *humungous*! What we do need to do is encourage our people to work on original areas to give them the best shot of having a job in academia – if that's what they want to do – but also be honest with them that another path could make them happier. The post-docs who come from foreign countries are in a genuinely tough spot as they may not have job opportunities back home, and I would love to find easier ways to provide support to incorporate them in the academic job market in the US.

**That's a thoughtful point. To finish up, if not science, what career would you have chosen? You can't say medicine.**

Oh I can't say medicine? I really like medicine. OK, I wanted to be a marine biologist when I was little. So Jacques Cousteau was one of my heroes. But I never learnt to dive; that's on my bucket list now.

**What do you do for fun at Cold Spring Harbor?**

I have a family who keeps me balanced and I've taken up fishing as my hobby here. I've concluded that I could never become a professional fisherman because I don't catch enough fish, but I really love being out on the water. Cold Spring Harbor is one of the most beautiful spots on the planet and so I'm blessed to be here.

**What one thing would people be surprised to learn about you?**

I'm a huge [Chicago] Cubs fan - that's something that not many people know because I'm too embarrassed to wear my Cubs hat! I wore it once on a holiday in the Caribbean and people stopped me and said, ‘I respect you, but I feel real sorry for you’. I thought, I work on pancreas cancer and here are people feeling sorry for me based on the sports teams that I like! But we did finally win the World Series this year, after 108 years. It will take us less time to beat pancreatic cancer!

